# Pediatric Abusive Head Trauma: A Systematic Review

**DOI:** 10.3390/diagnostics11040734

**Published:** 2021-04-20

**Authors:** Aniello Maiese, Francesca Iannaccone, Andrea Scatena, Zoe Del Fante, Antonio Oliva, Paola Frati, Vittorio Fineschi

**Affiliations:** 1 Department of Surgical Pathology, Medical, Molecular and Critical Area, Institute of Legal Medicine, University of Pisa, 56126 Pisa, Italy; aniello.maiese@unipi.it (A.M.); friann89@gmail.com (F.I.); a.scatena2@studenti.unipi.it (A.S.); 2 IRCSS Neuromed Mediterranean Neurological Institute, Via Atinense 18, 86077 Pozzilli, Italy; paola.frati@uniroma1.it; 3 Department of Anatomical, Histological, Forensic and Orthopaedic Sciences, Sapienza University of Rome, Viale Regina Elena 336, 00161 Rome, Italy; zoe.delfante@uniroma1.it; 4 Department of Health Surveillance and Bioethics, Section of Legal Medicine, Catholic University, Fondazione Policlinico A. Gemelli IRCCS, 00100 Rome, Italy; antonio.oliva@unicatt.it

**Keywords:** abusive head trauma, post-mortem CT, forensic pathology, shaken baby syndrome, retinal hemorrhage

## Abstract

Abusive head trauma (AHT) represents a commonly misdiagnosed condition. In fact, there is no pathognomonic sign that allows the diagnosis in children. Therefore, it is such an important medico-legal challenge to evaluate reliable diagnostic tools. The aim of this review is to evaluate the current scientific evidence to assess what the best practice is in order to diagnose AHT. We have focused particularly on evaluating the importance of circumstantial evidence, clinical history, the use of postmortem radiological examinations (such as CT and MRI), and the performance of the autopsy. After autopsy, histological examination of the eye and brain play an important role, with attention paid to correlation with symptoms found in vivo.

## 1. Introduction

Child abuse is the physical, sexual, and/or psychological abuse or neglect of a child or children, especially by a parent or caregiver. This condition can include any act or omission by a parent or caregiver that results in actual or potential harm to a child.

Typically, four categories of abuse can be distinguished: physical abuse, sexual abuse, emotional/psychological abuse, and neglect. More specifically, physical abuse can occur through hitting, beating, kicking, shaking, biting, choking, scalding, burning, poisoning, and suffocating.

Over the years, several classifications of physical abuse have been proposed.

Although shaken baby syndrome (SBS) has been the most commonly used definition in recent decades, abusive head trauma (AHT) has now been proposed as the most accurate [1].

While SBS refers only to shaking-related brain injury, AHT encompasses numerous different mechanisms of non-accidental traumatic injury to a child’s head.

More specifically, AHT is defined as an injury to the skull or intracranial contents of an infant or young child (<5 years of age) from a blunt blow and/or violent shaking [2].

Traditionally, a diagnostic clinical triad was seen in the simultaneous occurrence of subdural hematoma, retinal hemorrhage, and encephalopathy.

Recently, however, several authors have suggested that the signs recognized by the triad are not pathognomonic and should be correctly distinguished from the mechanism of injury (violent shaking). Victims of AHT often present with findings related to brain injury. Depending on the severity of the injury and the developmental age of the child, these symptoms may be obvious and pronounced or subtle and nonspecific. In fact, infants and younger children often have nonspecific symptoms, such as vomiting, that can be misinterpreted or even overlooked by clinicians [3]. A red flag for maltreatment is a history presented by the child’s caregivers that is incomplete or even contradicts clinical findings (e.g., a history of a brief fall that resulted in a clinically significant intracranial injury) [2,3,4].

To find out how current medical practice identifies AHT, we focused our attention on examining the symptom triad. We sought to analyze the relevant studies using objective criteria to identify the behaviors that may define best practice in diagnosing AHT. Finally, aware of the inherent limitations determined by a variety of factors, both instrumental and circumstantial, we made a comparison with a case study that proved emblematic.

## 2. Materials and Methods

### 2.1. Eligibility Criteria

The present systematic review was carried out according to the Preferred Reporting Items for Systematic Review (PRISMA) standards [5]. We used an evidence-based model for framing a PICO.

### 2.2. Search Criteria and Critical Appraisal

A systematic literature search and a critical appraisal of the collected studies were conducted. An electronic search of PubMed, Science Direct Scopus, and Excerpta Medica Database (EMBASE) from the inception of these databases to 10 February 2021 was performed. The search terms were “Abusive head trauma”, “Shaken infant syndrome”, “Shaken baby syndrome”, and “Retinal hemorrhage” in the title, abstract and keywords. Bibliographies of all identified documents were reviewed and compared for further relevant literature. Methodological evaluation of each study was conducted according to the PRISMA standards, including assessment of bias. Data collection involved study selection and data extraction. Three researchers (A.M., A.O., P.F.) independently reviewed those documents whose title or abstract appeared to be relevant and selected those which analyzed the miRNAs. PMI eligibility between the researchers was resolved by a consensus process. No unpublished or grey literature was searched. Data extraction was performed by one investigator (A.M., A.S., F.I.) and verified by another investigator (V.F., Z.D.F.). Only papers or abstracts in English were included in the search.

## 3. Results

### 3.1. Search Results and Included Studies

An appraisal based on titles and abstracts, as well as a hand search of reference lists, were carried out. The reference lists of all located articles were reviewed to detect still unidentified literature. This search identified 4790 articles, that were then screened based on their abstract to identify their relevance in respect to the following:

- Estimate diagnosis process;
- Clinical features analyzed;
- Circumstantial data evaluation;
- Post-mortem evaluation;
- Study design.

**Figure 1:**
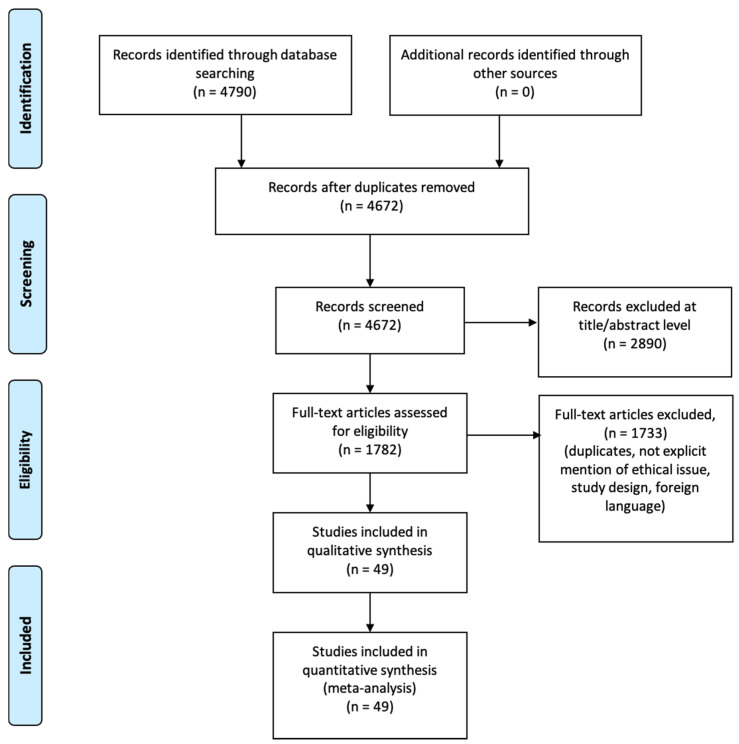
Preferred Reporting Items for Systematic Review (PRISMA) flow chart—search strategy. Study designs comprised retrospective and prospective studies, original articles, and reviews. An appraisal based on titles and abstracts as well as a hand search of reference lists were carried out. The reference lists of all located articles were reviewed to detect still unidentified literature. A total of 49 studies fulfilled the inclusion criteria.

The methodology of our search strategy is represented in Figure 1.

A further categorization of the articles included in the study was made on the basis of the main parameter under study, as showed by the following table (Table 1).

### 3.2. Risk of Bias

This systematic review has a number of strengths that include the amount and breadth of the studies, which span the globe; the hand search and scan of reference lists for the identification of all relevant studies; and a flowchart that describes in detail the study selection process. It must be noted that this review includes studies that were published in a time frame of 10 years; thus, despite our efforts to fairly evaluate the existing literature, study results should be interpreted taking into account that the accuracy of the clinical procedures, where reported, has changed over the years.

## 4. Discussion

The differential diagnosis between AHT and other mimic medical or surgical conditions (accidental traumatic brain injury, cerebral sino-venous thrombosis, hypoxic-ischemic injury) is a common dilemma in medical and legal practice because the severity of the consequences of either a false-positive or false-negative diagnosis requires the establishment of precise diagnostic criteria. Contemporary AHT diagnosis is based on a thorough clinical, radiological, and ophthalmological evaluation of the so-called “classic triad” (SDH, encephalopathy and RHs) as well as other injuries consistent with the mechanism of trauma.

The aim of our study was to evaluate the best diagnostic protocol by reviewing the literature and providing a case in which this protocol was correctly applied, to reduce the rate of misdiagnosed or criminal cases that end without a verdict, due to “lack of sufficient suspicion”, as shown by Feld et al. [53].

### 4.1. Clinical Findings

Maltreated infants and children may have findings ranging from nonspecific symptoms requiring only supportive care to acute life-threatening complications requiring urgent treatment (respiratory failure, severe respiratory distress, intracranial hypertension, decreased consciousness, seizures, and shock). Symptoms are variable and depend on the duration and intensity of the shaking, the age and constitution of the child and the offender [10].

The main consequence is that healthcare providers may initially misdiagnose or delay the diagnosis of pediatric AHT, leading to repeated episodes of inflicted trauma. In addition, caregivers often delay carrying the child to treatment until their condition is critical, resulting in a worse short- and long-term prognosis; indeed, although most victims survive, the mortality rate ranges from 13% to 35%, and only 10–15% of survivors have little to no impairment or disability (most commonly, visual deficit, hearing loss, and cognitive impairment) [8]. 

However, some signs and symptoms, although non-specific, have proven to be “red flags” for AHT: irritability/lethargy, altered mental status, respiratory impairment, multiple fractures (especially when in different stages of healing), varying degrees of abrasions or bruising in unusual locations (especially when observed in a non-mobile child), vomiting, and poor nutrition [11,47].

The “classic” symptomatologic triad of subdural hematoma (SDH), cerebral edema, and retinal hemorrhage (RH) remains a useful diagnostic tool; however, its absence should not lead the clinician to rule out AHT, because such symptoms may be absent in approximately 20% of cases [13].

SDH is a cardinal intracranial finding associated with AHT; therefore, the presence of SDH in an infant with inadequate history has a high association with AHT [8].

#### 4.1.1. Radiology

Skeletal injuries, particularly rib fractures and fractures of the long bones (most commonly the humerus and femur), have also been repeatedly noted in association with AHT in infants, resulting from compression of the infant’s chest during shaking or beating. Diaphyseal fractures of the long bones are strongly associated with inflicted trauma (physical abuse) in children <1 year of age, whereas accidental mechanisms become more common with increasing age [12].

Neuroimaging features more commonly associated with AHT include epidural hematoma, intraparenchymal injury, and skull fracture [16].

After performing standard laboratory tests (complete blood count with platelet count, chemistry panel, coagulation tests) to rule out other diagnoses, imaging studies are appropriate because they are the most important tests to confirm AHT. Clinicians should perform a skeletal examination (plain radiographs of the skull, spine, ribs, and long bones) and brain ultrasound or head CT, preferably unenhanced. In fact, in most institutions, a head examination CT is usually the first screening examination for suspected traumatic brain injury because it is very sensitive to skull fractures and intracranial hemorrhage as well as cerebral edema and ischemic changes; however, the use of ultrafast screening MRI protocols is gradually becoming routine for further evaluation of all patients with abnormal findings on screening examinations and for all patients with normal findings on CT, but with persistent clinical concerns. Unsuspected spinal lesions are found in up to 75% of patients with AHT; therefore, MRI of the cervical spine should be routinely performed along with brain imaging [23,24,25].

Clinical history is often unreliable; therefore, neuroimaging is also important in order to obtain information about the timing of the injury. Compared to CT, age determination of SDH can be estimated with better accuracy with MRI, especially for small blood collections, thus making magnetic resonance imaging (MRI) the most sensitive method of assessing the brain, particularly in brain-injured patients, because it allows visualization of blood degradation residues (BDR) in T2-weighted (T2W) images, although loss of detectability of hemosiderin in both sequences has been demonstrated in 10–20% of cases one year after trauma. A relatively recent development in MRI sequencing, susceptibility-weighted imaging (SWI), which is a three-dimensional, high-resolution gradient echo sequence with complete flow compensation, enables the detection of BDR even one year after trauma; therefore, it is rapidly becoming part of the brain MRI protocol, especially in brain-injured patients or patients with previous or suspected intracranial hemorrhage [14].

**Figure 2:**
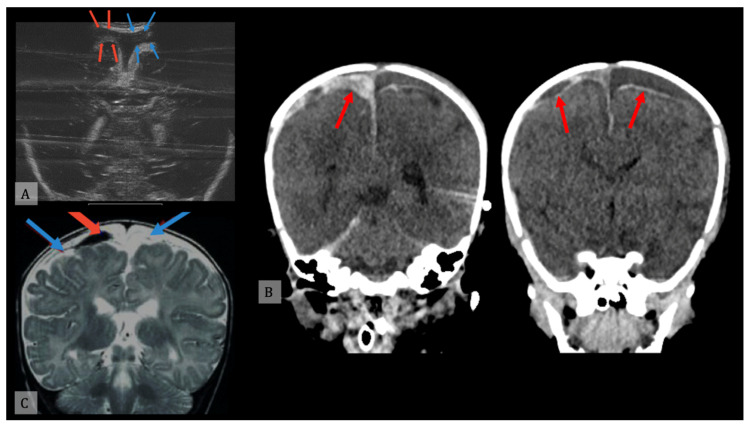
(**A**) Ultrasound examination of subdural (red arrows) and subarachnoid (blue arrows) hemorrhage. (**B**) Head computed tomography of subdural hemorrhage (red arrows). (**C**) Head magnetic resonance imaging of subdural (red arrow) and subarachnoid (blue arrows) hemorrhage.

DWI is the most sensitive sequence in detecting early parenchymal acute cytotoxic edema, hours before any changes appear on T2-FLAIR images, and days before CT; it can also identify new areas of brain injury weeks after initial injury, and more extensive damage compared to conventional T2-FLAIR images [54] (Figure 2).

#### 4.1.2. Ophthalmological Examination

RH is exceptionally rare in the absence of traumatic head injury; therefore, RH remains the most reliable clinical feature of the triad: a U.K. study by Maguire et al. found that RH was seen on ophthalmic examination in 78% of AHT cases (compared with 5% of accidental head injuries) [17,18].

RH has been described in children with accidental trauma, such as car accidents or critical illness, but RH from accidental causes typically occurs in a pattern markedly different from those associated with AHT: extensive (too numerous to count), bilateral hemorrhages extending to the outer edges of the retina (ora serrata retinae) and involving multiple layers, although not pathognomonic, are strongly associated with AHT. Furthermore, the severity of RH is directly related to the severity of brain injury in children with traumatic brain injury, as shown in the work of Bibenbaum and colleagues [19,20,36,55].

Furthermore, RH has been described, although rarely, to be present in victims of AHT without radiographic signs of intracranial injury at neuroimaging [21,22].

In a report of child victims of AHT by Vinchon and colleagues, the combination of SDH, severe RH, and the absence of findings of head impact had a sensitivity of 0.24 but a specificity of 1.0; thus, the presence of such findings in combination is quite specific, but their absence is not a good indicator for ruling out child abuse [18].

The estimated incidence of RHs in maltreated children is up to 75%; 85% according to the work of Levin and colleagues, which also showed that the incidence of RH in children who died as a result of AHT is 10-fold higher than in non-maltreated survivors, pointing to RH severity as a crucial prognostic factor [22,48].

Although many pathologic conditions can condition RHs, severe, bilateral, multilevel RHs or optic nerve sheath hemorrhages (typically described in AHTs) are seen only in patients with severe coagulopathy, sepsis, and advanced-stage myeloid leukemia [26].

Given the importance of timely recognition of RHs, the intervention of an ophthalmologist should be requested in any case of suspected AHT, even if other health professionals are experienced in ophthalmic examination. Increasing RH severity correlates with increasing risk of AHT; therefore, RH, identified on MRI, must be considered with suspicion of AHT. Without a history of severe trauma, the discovery of a signal intensity abnormality in the posterior orbits in children should prompt additional investigation for intracranial injury.

Dilated fundoscopic examination (DFE) is the reference standard; a CT-scan or MRI may be performed as a second-level work-up. In CT-scans, RHs appear as punctate foci with high density in the posterior globes and as foci with low signal intensity on MRI. Beavers et al. reported 61% sensitivity for confirmed RH with simultaneous analysis of T2*-weighted, T2-weighted, FLAIR, T1-weighted, and T1-weighted contrast-enhanced sequences, with decreasing sensitivity of sequences in the order listed. They also correlated funduscopic severity and MRI detection rates and found that 76% of high-grade RHs were detected on MRI compared with only 14% of low-grade RHs [27].

A study by Zuccoli et al. showed that SWI (susceptibility weight imaging) ocular MRI has a sensitivity of 80% in detecting RHs compared to the 62% of standard MRI, suggesting that a high-resolution orbit SWI protocol should be the gold standard in identifying and assessing such hemorrhages [28]. However, because MRI is not a routine imaging modality, a DFE performed by an experienced ophthalmologist remains the diagnostic gold standard.

Imaging of retinal hemorrhages in suspected abusive head trauma is an essential part of the documentation. Previously, fundus cameras coupled to digital indirect ophthalmoscopes or panretinal imaging with contact of the camera with the cornea have been used. Recently, however, findings from handheld spectral-domain optical coherence tomography in shaken baby syndrome have been reported. The authors focused detection on the vitreo-retinal interface rather than all hemorrhages present and demonstrated the lack of consistency of the clinical classification of RetCam images of pediatric RHs based on the commonly held defining features of hemorrhages in different retinal layers due to significant inter- and intraobserver variability [29].

### 4.2. Forensic Pathology

#### 4.2.1. Post-Mortem CT

**Figure 3:**
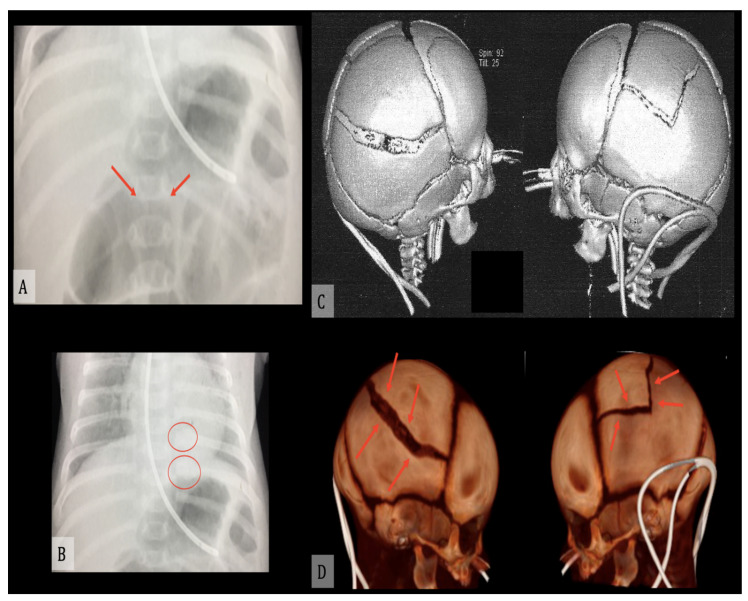
(**A**) Radiography of D11 soma fracture (red arrows). (**B**) Radiography of VII and VIII left rib fractures (red circles). (**C**,**D**) Three-dimensional CT reconstruction of skull fractures (red arrows).

Radiology is a valid diagnostic tool not only to diagnose AHT in vivo, but also post-mortem, and its use in the forensic investigation, as either a supplement or an alternative to traditional autopsy, is constantly increasing. Among its numerous advantages are it being non-destructive, unlike dissection, and the possibility to retain a permanent record of the findings; moreover, it is effective in the identification of fractures and internal hemorrhages, the most common findings in AHT cases (Figure 3).

As proposed by Stein, in order to efficiently locate ruptured vases without damaging them when extracting and cutting the brain, a post-mortem unenhanced CT of the infant’s head to document its original state could first be performed, and then repeated after instilling contrast agent directly via fontanel puncture into the superior sagittal sinus. This method is minimally invasive and can be conducted quickly and conveniently on clinical CT systems, but it requires open fontanels [56].

#### 4.2.2. Autopsy 

The autopsy exam must be focused on detecting external and internal signs compatible with abuse. It also should be carried out paying particular attention to a complete anamnestic, circumstantial, and clinical collection, in order to verify the correspondence of such data with the autopsy findings. During the autopsy, it is mandatory to take the entire brain, to preferably examine it after formalin fixation. 

The most common finding at autopsy of inflicted head trauma is subdural hemorrhage (SDH), mostly associated with multiple cranial fractures, which is found in about 90% to 98% of such cases. The most typical form of SDH at autopsy in an inflicted head injury is a thin layer of SD blood over the cerebral convexities, one or both (more commonly both), and the amount of blood may be less than 10 mL. Another common finding is subarachnoid hemorrhage (SAH), caused by rotational inertial brain injury in which the tearing of bridging veins—which pass through the arachnoid membrane to reach the dural sinuses—occurs. In cases of inflicted neurotrauma, the typical appearance is small patches of SAH along the cerebral parasagittal convexities. Thus, investigation for bridging vein rupture should be a routine post-mortem procedure in cases of suspect head trauma: a finding of intact bridging veins can rule out trauma as the cause of an eventual subdural bleeding, while the finding of bridging vein rupture definitively identifies mechanical trauma as the cause of a child’s death [57].

It is also advisable to remove one or both eyes for detailed examination after fixation in formaldehyde; following fixation, the eyes are examined grossly and then sectioned into segments that allow visualization of the interior of the eye. The removal of the eye can be performed via an anterior approach, appropriate when it is not required to examine orbital structures other than the eye, or a posterior approach, that should be preferred when it is necessary to remove the globe in continuity with the other orbital contents; in either approach, the first step is the incision of the conjunctiva. To achieve an adequate cosmetic result, the orbit must be reconstructed right after the removal of the eye, and great care must be taken not to damage the eyelids or eyelashes when removing the eye globes [57].

**Figure 4:**
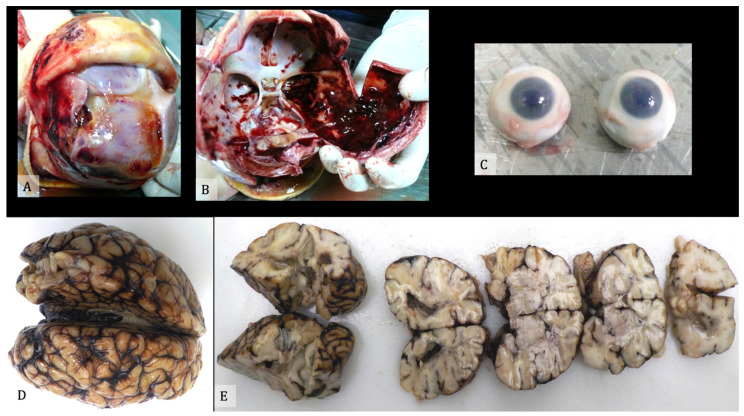
(**A**) External view of the skull after scalp removal. (**B**) Internal cranial cavity. (**C**) Eyes samples. (**D**) Brain after formalin embedding. (**E**) Brain slices.

All findings should be documented and photographed, and samples from each organ should be taken for microscopic examination. The findings should be described and documented as to the presence of optic nerve sheath hemorrhage, retinal hemorrhages, and the presence of any schisis of the retina or hemorrhage in the vitreous. Descriptions of any retinal hemorrhages should include notation of the locations of the hemorrhages (posterior, periphery, or reaching the ora serrata), the number (few, many, or too numerous to count) and which of the retinal layers are affected. The optic nerve sheath must be examined closely to determine whether there is hemorrhage within it and/or the nerve [58] (Figure 4).

However, Breazzano et al. also found that in younger children, who are more susceptible to damage from traction of the vitreomacular apparatus caused by rotation or acceleration/deceleration, any attempt to identify AHT-induced hemorrhage results in irreversible damage to the macula and optic nerve [51].

#### 4.2.3. Histology

When clinical examination is followed by postmortem ocular examination, significant discrepancy between clinical diagnosis and autoptic findings is frequently reported. Histopathologic examinations in cases of retinal folds in childhood have demonstrated hemorrhagic schisis beneath the internal limiting membrane, subhyloid hemorrhage, and subretinal hemorrhage, as described in the work of Squier et al. [7,51].

**Figure 5:**
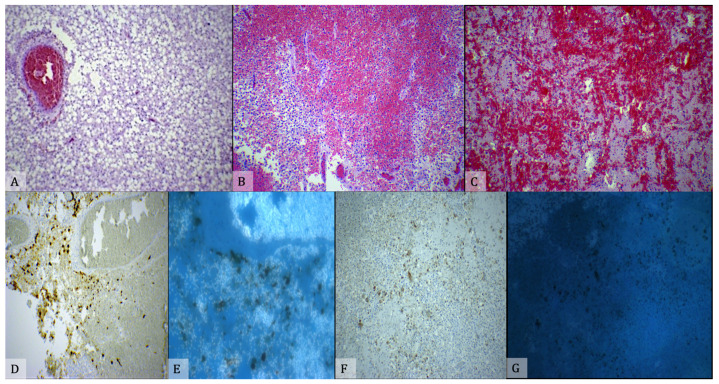
(**A**) Extensive parenchymal vacuolization with optically empty vacuoles surrounding a predominantly glial cellularity. (**B**) Infiltration of the parenchyma by large collections of erythrocytes surrounded by brain tissue dominated by glial reaction. (**C**) Disappearance of the parenchyma with replacement by extensive erythrocyte lakes. (**D**,**E**) Immunohistochemical examination with reaction to GFAP antibody shows strong parenchymal positivity, which can be graded 4+ using a semi-quantitative scale. (**E**) The specimens were examined by means of a confocal microscope (True Confocal Scanner, Leica TCS SP2). (**F**,**G**) The antibody response for macrophages (CD68+) is strongly positive and occupies large parenchymal areas in many fields. (**G**) The specimens were examined by means of a confocal microscope (True Confocal Scanner, Leica TCS SP2).

An immunohistochemical process using beta-amyloid precursor protein staining (βAPP) will allow damaged axons to be seen as early as 2 h after damage. The interpretation of the immunohistochemical staining with βAPP requires understanding that a variety of patterns of axonal damage exists, including damage from trauma (tDAI), hypoxic/ischemic insult (vDAI), and metabolic insult (mDAI) [59,60]. Another useful immunohistochemical marker is Glycophorin-A, an erythrocyte membrane protein, whose positivity confirms the presence of an area of hemorrhagic spread (Figure 5).

**Figure 6:**
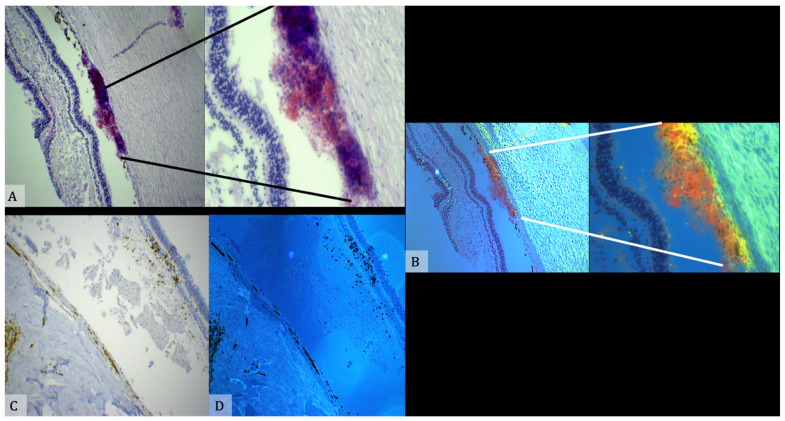
(**A**) The retina shows a well-preserved retinal plane with congested vessels on section surface. Immediately below the retina, an extravasation of erythrocytes is visible adherent to the choroid. (**B**) The same sample was analyzed by an immunohistochemical method sensitive to Glycophorin. (**C**,**D**) Reaction to Glycophorin A antibody shows positivity in the subretinal area. (**B**,**D**) The specimens were examined by means of a confocal microscope (True Confocal Scanner, Leica TCS SP2).

Histological analysis of eye samples is most important in such cases, because it could confirm the diagnosis by showing the presence of: intraretinal hemorrhages (particularly the so called “cherry hemorrhages”), intrascleral hemorrhages, subdural hemorrhages in the optic nerve sheath and perimacular folds, all considered specific findings for abusive head trauma in the appropriate clinical situation—although not pathognomonic—because of their strong correlation to high-acceleration traumas. The identification of cherry hemorrhages in the internal limiting membrane (ILM) is one of the most robust criteria for identifying abusive head trauma, because they are found only in eyes that have a torn ILM and concurrent retinal hemorrhages extending to the ora serrata, both microscopic findings associated with AHT [51]. This showed well-preserved retinal layers and congested vessels. Erythrocyte leakage was noted below the retina and strongly adherent to the choroid. The same region showed strong positivity for glycophorin A antibodies (Figure 6).

## 5. Conclusions

AHT represents a major cause of fatal head injury in infants and young children, but the diagnosis is not straightforward and represents a medical, forensic, and social challenge, achievable only through a multidisciplinary approach. The diagnosis is primarily based on an accurate clinical history, a thorough ophthalmological examination (eye fundus) and neuroimaging to detect signs of brain and spinal injury [61]. Retinal hemorrhages are a reliable means of proving the diagnosis AHT both in vivo and postmortem; therefore, in case of postmortem evaluation, histological examination of the retinal layer could allow confirmation of the clinical diagnostic hypothesis.

In conclusion, through a careful analysis of the literature, we can state that not all studies agree on the necessity of the classical triad for diagnoses of AHT; while the in vivo diagnostic process is shared for most cases, there appears to be an all-encompassing protocol for post-mortem diagnosis; we have therefore attempted to develop a single diagnostic pathway that brings together all the tools currently available to the forensic pathologist.

## Figures and Tables

**Table 1:** Categorization of the articles included in the study on the basis of the main parameter analyzed.

Author	Title	Clinical History	Radiological Evaluation of the Brain and/or CNS	Ophthalmologic Evaluation	All Items
Minns et al. [6]	Prediction of inflicted brain injury in infants and children using retinal imaging			X	
Squier [7]	The “Shaken Baby” syndrome: Pathology and mechanisms				X
Parks et al. [8]	Characteristics of non-fatal abusive head trauma among children in the USA, 2003–2008: Application of the CDC operational case definition to national hospital inpatient data				X
Levin and Christian [9]	The eye examination in the evaluation of child abuse			X	
Bartschat et al. [10]	Long-term outcome in a case of shaken baby syndrome	X			
Hung [11]	Pediatric abusive head trauma	X			
Yu et al. [12]	Injury patterns of child abuse: experience of two level 1 pediatric trauma centers				X
Mian et al. [13]	Shaken baby syndrome: A review				X
Schelhorn et al. [14]	Intracranial hemorrhage detection over time using susceptibility-weighted magnetic resonance imaging		X		
Adamsbaum et al. [15]	Dating the abusive head trauma episode and perpetrator statements: Key points for imaging	X	X		
Wootton-Gorges et al. [16]	ACR appropriateness criteria^®^ suspected physical abuse-child	X	X		
Maguire et al. [17]	Retinal haemorrhages and related findings in abusive and non-abusive head trauma: A systematic review	X		X	
Vinchon et al. [18]	Confessed abuse versus witnessed accidents in infants: Comparison of clinical, radiological, and ophthalmological data in corroborated cases				X
Binenbaum et al. [19]	Retinal hemorrhage and brain injury patterns on diffusion-weighted magnetic resonance imaging in children with head trauma		X	X	
Christian et al. [20]	The eye examination in the evaluation of child abuse			X	
Binenbaum et al. [21]	Patterns of retinal hemorrhage associated with increased intracranial pressure in children			X	
Bhardwaj et al. [22]	A systematic review of the diagnostic accuracy of ocular signs in pediatric abusive head trauma			X	
Choudhary et al. [23]	Consensus statement on abusive head trauma in infants and young children				X
Elinder et al. [24]	Traumatic shaking: The role of the triad in medical investigations of suspected traumatic shaking				X
Bradford et al. [25]	Serial neuroimaging in infants with abusive head trauma: Timing abusive injuries		X	X	
Agrawal et al. [26]	Prevalence of retinal hemorrhages in critically ill children			X	
Gekat et al. [27]	SDH and EDH in children up to 18 years of age—A clinical collective in the view of forensic consideration		X	X	
Zuccoli et al. [28]	Susceptibility weighted imaging depicts retinal hemorrhages in abusive head trauma			X	
Mulvihil et al. [29]	An inter-observer and intra-observer study of a classification of RetCam images of retinal haemorrhages in children			x	
Barnes et al. [30]	Infant acute life-threatening event—Dysphagic choking versus nonaccidental injury	X	X		
Miller et al. [31]	The significance of macrocephaly or enlarging head circumference in infants with the triad: Further evidence of mimics of shaken baby syndrome		X		
Carrim et al. [32]	Presumed non-accidental injury with retinal haemorrhages—Findings from a tertiary referral centre in the United Kingdom			X	
Adamsbaum et al. [33]	Abusive head trauma: Judicial admissions highlight violent and repetitive shaking	X	X		
Kemp et al. [34]	Neuroimaging: What neuroradiological features distinguish abusive from non-abusive head trauma? A systematic review		X		
Maguire et al. [35]	Estimating the probability of abusive head trauma: A pooled analysis				X
Wu et al. [36]	Pediatric abusive head trauma in Taiwan: Clinical characteristics and risk factors associated with mortality				X
Babl et al. [3]	Pediatric abusive head trauma in the emergency department: A multicentre prospective cohort study		X		
Fraser et al. [37]	Prevention and recognition of abusive head trauma: Training for healthcare professionals in Vietnam	X			
Loredo-Abdalá et al. [38]	Pediatric abusive trauma: Multicentric experience in Mexico				X
Ferguson et al. [39]	Abusive head trauma and mortality: An analysis from an international comparative effectiveness study of children with severe traumatic brain injury	X			
Thamburaj et al. [40]	Susceptibility-weighted imaging of retinal hemorrhages in abusive head trauma			X	
Andersson and Thiblin [41]	National study shows that abusive head trauma mortality in Sweden was at least 10 times lower than in other Western countries	X	X		
Morgan et al. [42]	Clinical comparison of ocular and systemic findings in diagnosed cases of abusive and non-abusive head trauma		X	X	
Gencturk et al. [43]	Various cranial and orbital imaging findings in pediatric abusive and non-abusive head trauma, and relation to outcomes		X	X	
Sidpra et al. [44]	Skull fractures in abusive head trauma: A single centre experience and review of the literature		X		
Wright et al. [45]	Disability and visual outcomes following suspected abusive head trauma in children under 2 years			X	
Donaldson et al. [46]	Ophthalmology referral as part of a multidisciplinary approach to suspected abusive head trauma			X	
Payne et al. [47]	Recognition and nursing management of abusive head trauma in children	X	X		
Levin [48]	Retinal hemorrhage in abusive head trauma			X	
Drubach et al. [49]	Skeletal trauma in child abuse: Detection with 18F-NaF PET		X		
Laghmari et al. [50]	Birth-related retinal hemorrhages in the newborn: incidence and relationship with maternal, obstetric and neonatal factors. Prospective study of 2,031 cases				
Breazzano et al. [51]	Clinicopathological findings in abusive head trauma: analysis of 110 infant autopsy eyes			X	
Piteau et al. [52]	Clinical and radiographic characteristics associated with abusive and nonabusive head trauma: A systematic review	X	X		

## Data Availability

Data available on request due to restrictions e.g., privacy or ethical reason.
